# Sodium Intake and Cause-Specific Mortality Among Predominantly Low-Income Black and White US Residents

**DOI:** 10.1001/jamanetworkopen.2024.3802

**Published:** 2024-03-26

**Authors:** Hyung-Suk Yoon, Qiuyin Cai, Jae Jeong Yang, Loren Lipworth, Hui Cai, Danxia Yu, Mark D. Steinwandel, Deepak K. Gupta, William J. Blot, Wei Zheng, Xiao-Ou Shu

**Affiliations:** 1 Vanderbilt-Ingram Cancer Center, Vanderbilt Epidemiology Center, Division of Epidemiology, Department of Medicine, Vanderbilt University School of Medicine, Nashville, Tennessee; 2 University of Florida Health Cancer Center, University of Florida, Gainesville; 3 Department of Surgery, College of Medicine, University of Florida, Gainesville; 4 Vanderbilt Translational and Clinical Cardiovascular Research Center, Vanderbilt University Medical Center, Nashville, Tennessee; 5 International Epidemiology Field Station, Vanderbilt Institute for Clinical and Translational Research, Rockville, Maryland; 6 Division of Cardiovascular Medicine, Vanderbilt University Medical Center, Nashville, Tennessee

## Abstract

**IMPORTANCE:**

Epidemiologic evidence regarding the outcomes of dietary sodium intake on mortality remains limited for low-income individuals, particularly Black people.

**OBJECTIVE:**

To investigate the associations of excessive dietary sodium with all-cause and cause-specific mortality among predominantly low-income Black and White Americans.

**DESIGN, SETTING, AND PARTICIPANTS:**

This cohort study included participants aged 40 to 79 years from the Southern Community Cohort Study who were recruited at Community Health Centers in 12 southeastern states from 2002 to 2009. Analyses were conducted between March 2022 and June 2023.

**EXPOSURES:**

Dietary sodium intake was assessed using a validated food frequency questionnaire at baseline.

**MAIN OUTCOMES AND MEASURES:**

Multivariable-adjusted Cox regression was used to estimate hazard ratios (HRs) and 95% CIs for mortality outcomes (all-cause, cardiovascular disease [CVD], coronary heart disease [CHD], stroke, heart failure, cancer, and other) associated with sodium intake. Nonlinear associations and population-attributable risk (PAR) of the mortality burden associated with excess sodium were further assessed.

**RESULTS:**

Among the 64 329 participants, 46 185 (71.8%) were Black, 18 144 (28.2%) were White, and 39 155 (60.9%) were female. The mean (SD) age at study enrollment was 51.3 (8.6) years for Black participants and 53.3 (9.3) years for White counterparts. Mean (SD) dietary sodium intake was 4512 (2632) mg/d in Black individuals and 4041 (2227) mg/d in White individuals; 37 482 Black individuals (81.2%) and 14 431 White individuals (79.5%) exceeded the current dietary recommendations of 2300 mg/d. During a median (IQR) follow-up of 13.8 (11.3-15.8) years, 17 811 deaths were documented, including 5701 from CVD. After adjustment for potential confounders, in Black individuals, HRs per 1000-mg increase in daily sodium intake were 1.07 (95% CI, 1.03-1.10) and 1.08 (95% CI, 1.02-1.14) for deaths from total CVD and CHD, respectively; while in White individuals, the corresponding HRs were 1.08 (95% CI, 1.02-1.14) and 1.13 (95% CI, 1.03-1.23). No significant associations were found for cancer mortality. PAR estimates suggest that sodium intake above the recommended threshold may account for 10% of total CVD, 13% of CHD, and 30% of heart failure deaths in this low-income southern population.

**CONCLUSIONS AND RELEVANCE:**

In this cohort study of 64 329 low-income Americans, nearly 80% of study participants consumed sodium exceeding the current recommended daily amount, which was associated with 10% to 30% of CVD mortality. Public health programs targeted to reduce sodium intake among this underserved population may be beneficial.

## Key Points

**Question:**

How is sodium intake associated with mortality outcomes among low-income Black and White Americans?

**Findings:**

This cohort study of 64 329 Americans from the Southern Community Cohort Study found that approximately 80% of low-income Black and White Americans exceeded the recommended daily intake of sodium, which was associated with 10% to 30% of cardiovascular disease mortality.

**Meaning:**

These findings suggest that approaches to lower sodium intake among underserved populations need to be established as the primary public health priority.

## Teaser

This cohort study evaluates the associations of excessive dietary sodium with all-cause and cause-specific mortality among predominantly low-income Black and White US residents.

## Introduction

Sodium is an essential nutrient for normal physiologic function, but excessive intake is associated with adverse health outcomes.^1,2^ For health promotion and disease prevention, the 2020 to 2025 Dietary Guidelines for Americans recommend less than 2300 mg of sodium per day.^3^ Most US adults, however, consume 3400 mg per day, which far exceeds the body’s needs.^4^

Excessive sodium consumption has been linked to elevated blood pressure, a major risk factor for cardiovascular disease (CVD),^1,2^ and can directly affect multiple target organs and tissues, including the brain, heart, kidneys, and bones, contributing to increased mortality hazards.^5,6,7,8^ A report from the National Health and Nutrition Examination Survey (NHANES) found 46% higher CVD mortality among people with high sodium diets.^9^ In another NHANES study, high sodium intake accounted for the largest portion of estimated diet-related cardiometabolic deaths among Americans.^10^ A meta-analysis of 25 prospective studies reported a 16% increase in all-cause mortality associated with high sodium intake.^11^ A cohort study of elderly Americans also found higher mortality among those who exceeded the recommendation, although statistical significance disappeared after adjustment for confounders.^12^ Epidemiologic evidence to date supports the mortality burden due to excessive sodium intake.^2,13^ Nevertheless, this burden remains overlooked in marginalized populations, especially Black Americans and individuals with low socioeconomic status (SES), who consume more high-sodium foods than others.^14^

In this cohort study of low-income Black and White Americans, we explored the racial and socioeconomic differences in daily sodium consumption. Furthermore, we investigated the associations of sodium intake with all-cause and cause-specific mortality (ie, CVD and its subtypes, cancer, and other diseases) and estimated the burden of mortality attributable to excess dietary sodium among this underserved population.

## Methods

### Study Population

This study is based on the Southern Community Cohort Study (SCCS), a prospective cohort to investigate the root causes of longstanding racial and ethnic disparities in health.^15,16^ With written informed consent, approximately 85 000 adults aged 40 to 79 years were recruited in 12 southeastern US states (Alabama, Arkansas, Florida, Georgia, Kentucky, Louisiana, Mississippi, North Carolina, South Carolina, Tennessee, Virginia, and West Virginia) from 2002 to 2009. About 86% of study participants were recruited at Community Health Centers (CHCs) that provide primary health care services to low-income individuals, who form the basis of the current study. A baseline survey was performed to collect self-reported information on sociodemographics, lifestyle, diet, and medical history. Among participants enrolled at CHCs, we excluded participants who reported implausible energy intake (<600 or >8000 kcal/d) or who had invalid follow-up information. Participants who self-reported a race other than Black or White were excluded due to their small sample size. The SCCS protocol was approved by the institutional review boards at Vanderbilt University Medical Center and Meharry Medical College. This study followed the Strengthening the Reporting of Observational Studies in Epidemiology (STROBE) reporting guideline for cohort studies.

### Exposure Assessment

Dietary data were collected using a food frequency questionnaire (FFQ) at baseline. The SCCS FFQ, containing 89 food items, was developed to capture the usual diet for Black and White Americans living in the southeastern US.^17,18^ Intakes of total energy, macronutrients, and micronutrients, including sodium, were estimated using the US Department of Agriculture Food Composition Databases.^19^ The overall validity of the FFQ was evaluated using correlation coefficients between FFQ and multiple 24-hour dietary recalls: 0.59 to 0.83 for macronutrients and 0.43 to 0.81 for micronutrients.^19^ In this study, dietary sodium intake was classified into 5 categories: the recommended level (<2300 mg/d), above recommendation by up to 50% (2300-3450 mg/d), more than 50% to 100% or less (3451-4600 mg/d), more than 100% to 200% or less (4601-6900 mg/d), and over 200% (>6900 mg/d).

### Outcome Ascertainment

The survival status was ascertained by linking the Social Security Administration and the National Death Index through December 31, 2019. Underlying causes of death were defined using the *International Statistical Classification of Diseases and Related Health Problems, Tenth Revision*: total CVD, I00-I99; coronary heart disease (CHD), I20-I25; stroke, I60-I69; heart failure, I50-I50.9; cancer C00-C97; and other diseases excluding external causes.

### Statistical Analysis

Baseline characteristics across dietary sodium intake were compared using the χ^2^ test and the generalized linear model. Multivariable Cox proportional hazards regression was used to estimate hazard ratios (HRs) and 95% CIs for all-cause and cause-specific mortality, using the recommended level as the reference. Additionally, HRs and 95% CIs per 1000-mg increase in daily sodium intake were evaluated. Follow-up time was treated as the time scale, calculated as total years from the date of enrollment to the date of death or censoring (ie, loss-to-follow-up or latest follow-up). The global goodness-of-fit test with Schoenfeld residuals found no violation against the proportional hazards assumption. All analyses were conducted separately among Black and White individuals, given the potential racial differences. Under the conceptual framework established from the literature review (eFigure in Supplement 1), factors for adjustment were chosen based on their association with sodium intake and mortality, including age at enrollment (years), sex (men or women), education (completed up to high school or completed beyond high school), annual household income (<$25 000 or ≥$25 000), marital status (married or living alone), medical insurance (no or yes), smoking status (never, former, or current with ≤20 or >20 pack-years), physical activity (sex-specific tertiles of total metabolic equivalent hours), alcohol consumption (none, ≤28, or >28 g/d of ethanol in men; none, ≤14, or >14 g/d in women), body mass index (BMI, calculated as weight in kilograms divided by height in meters squared; <30 or ≥30), total energy intake (kcal/d), and healthy eating index (score).^20^ Missingness (mostly <1%) was handled with imputation using median or mode values of nonmissing covariates. Potential nonlinear associations between sodium intake and mortality were assessed by using restricted cubic spline regression, with the reference as 2300 mg/d of sodium and 3 knots fitted at the 5th, 50th, and 95th percentiles. Stratified analyses were conducted, and interactions between sodium intake and strata variables were tested by the likelihood ratio test using the multiplicative interaction term. Population attributable risks (PARs) and corresponding 95% CIs were calculated with the following equation^21^:

*PAR* = [*P* × (*RR*−1)]/{[*P* × (*RR*−1)]+1},

where *P* was the prevalence of dietary sodium intake, and RR was the relative risk (adjusted HRs in the current analyses).

A series of sensitivity analyses were performed by adjusting for history of hypertension or CVD and comorbidity index, excluding the first 2 years of observation, excluding individuals with history of CVD, and adopting competing risk models. All statistical analyses were conducted using SAS version 9.4 (SAS Institute), between March 2022 and June 2023. A 2-sided *P* value less than .05 was considered statistically significant.

## Results

This study included 64 329 participants with a mean (SD) age at study enrollment of 51.3 (8.6) years for Black participants and 53.3 (9.3) years for White counterparts; 46 185 (71.8%) were Black, 18 144 (28.2%) were White, 39 155 (60.9%) were female, and 82.8% reported annual household income less than $25 000. The mean (SD) dietary sodium intake was 4512 (2632) mg/d for Black participants and 4041 (2227) mg/d for White participants. Those with the lowest income level (annual household income <$15 000) consumed more sodium each day, with a mean (SD) 4593 (2695) mg/d for Black individuals and 4145 (2314) mg/d for White individuals (eTable 1 in Supplement 1). Sodium consumption exceeded the current 2300 mg/d recommendation in 37 482 (81.2%) and 14 431 (79.5%) Black and White adults, respectively; these individuals were more likely to be young, male, less educated, lower earners, current smokers, and heavy alcohol drinkers (Table 1). When stratified by race (eTable 2 in Supplement 1), Black individuals with a history of CVD were less likely to fall into the high salt intake group, but their White counterparts showed no differences in sodium intake. In addition, Black individuals appeared to show lower educational attainment, earned less, lived without a partner, drank heavily (more than 28 g/day for men or more than 14g/day for women), high energy intake (mean 2703 kcal/day for black individuals vs. 2309 kcal/day for White individuals), and were more likely to have obesity, hypertension, and diabetes than White individuals (eTable 3 in Supplement 1).

**Table 1.** Baseline Characteristics of Study Participants

Baseline characteristics	Dietary sodium intake, No. (%), mg/d^a^					*P* value
	<2300 (n = 12 416)	2300-3450 (n = 16 109)	3451-4600 (n = 12 651)	4601-6900 (n = 13 729)	>6900 (n = 9424)	
Enrollment age, mean (SD), y	53.7 (9.2)	53.1 (9.1)	51.9 (8.7)	50.7 (8.3)	48.8 (7.2)	<.001
Race						
Black Americans	8703 (70)	10 917 (68)	8943 (71)	10 073 (73)	7549 (80)	<.001
White Americans	3713 (30)	5192 (32)	3708 (29)	3656 (27)	1875 (20)	
Sex						
Men	2496 (20)	4453 (28)	4912 (39)	7252 (53)	6061 (64)	<.001
Women	9920 (80)	11 656 (72)	7739 (61)	6477 (47)	3363 (36)	
Education						
Completed up to high school	8251 (66)	10 315 (64)	8357 (66)	9282 (68)	6749 (72)	<.001
Completed education beyond high school	4165 (34)	5794 (36)	4294 (34)	4447 (32)	2675 (28)	
Annual household income						
<$15 000	7465 (60)	9337 (58)	7618 (60)	8533 (62)	6220 (66)	<.001
≥$15 000 to <$25 000	2661 (22)	3562 (22)	2807 (22)	3044 (22)	2046 (22)	
≥$25 000 to <$50 000	1539 (12)	2148 (13)	1569 (12)	1591 (12)	912 (10)	
≥$50 000	751 (6)	1062 (7)	657 (6)	561 (4)	246 (2)	
Marital status						
Married or living with a partner	3927 (32)	5635 (35)	4321 (34)	4358 (32)	2466 (26)	<.001
Living alone^b^	8489 (68)	10 474 (65)	8330 (66)	9371 (68)	6958 (74)	
Medical insurance	7634 (61)	9763 (61)	7326 (58)	7362 (54)	4580 (49)	<.001
Smoking status						
Never	5365 (43)	6417 (40)	4471 (35)	4003 (29)	2315 (25)	<.001
Former	2982 (24)	3845 (24)	2808 (22)	2639 (19)	1430 (15)	
Current with ≤20 pack-years	2351 (19)	3190 (20)	2953 (23)	3899 (28)	3358 (36)	
Current with >20 pack-years	1718 (14)	2657 (16)	2419 (19)	3188 (23)	2321 (25)	
Physical activity^c^						
Low	4955 (40)	5791 (36)	4221 (33)	4317 (31)	2447 (26)	<.001
Middle	4062 (33)	5473 (34)	4261 (34)	4342 (32)	2871 (30)	
High	3399 (27)	4845 (30)	4169 (33)	5070 (37)	4106 (44)	
Alcohol consumption^d^						
None	7257 (58)	8559 (53)	6056 (48)	5662 (41)	3256 (35)	<.001
Moderate	3996 (32)	5656 (35)	4539 (36)	4887 (36)	3296 (35)	
Heavy	1163 (9)	1894 (12)	2056 (16)	3180 (23)	2872 (30)	
Body mass index, mean (SD)	31.3 (7.5)	31.2 (7.6)	30.7 (7.7)	29.8 (7.5)	28.8 (7.3)	<.001
Healthy eating index, mean (SD)	60.5 (13.0)	58.8 (12.4)	56.6 (11.8)	55.1 (10.8)	54.2 (9.3)	<.001
Disease history						
Hypertension	7660 (62)	9694 (60)	7151 (57)	7168 (52)	4363 (46)	<.001
CVD	1601 (13)	2073 (13)	1498 (12)	1644 (12)	1014 (11)	<.001
Cause of death, No.						
All causes	3264	4331	3456	4011	2749	NA
Total CVD	1089	1392	1117	1227	876	NA
Cancer	736	942	796	944	614	NA
Other diseases	1233	1730	1299	1489	956	NA

Abbreviations: CVD, cardiovascular disease; NA, not applicable.

^a^ Mean (SD) intake was 4512 (2632) mg/d among Black individuals and 4042 (2227) mg/d among White individuals.

^b^ Included individuals who never married and those who were separated, divorced, widowed, or single.

^c^ Defined by tertiles of the total metabolic equivalent of task hours per week.

^d^ Defined as nondrinkers, 0 g/d; moderate drinkers, more than 0 but 28 or less g/d for men or more than 0 but 14 or less g/d for women; and heavy drinkers, more than 28 g/d for men or more than 14 g/d for women.

During a median (IQR) follow-up of 13.8 (11.3-15.8) years, 17 811 deaths were documented, including 5701 from CVD (2216 from CHD, 905 from stroke, 470 from heart failure, and 2110 from other), 4032 from cancer, and 6707 from other diseases. Compared with the recommended level of less than 2300 mg/d, higher sodium intake was associated with increased mortality (Table 2; eTable 4 in Supplement 1). Multivariable-adjusted HRs associated with intake greater than 6900 mg/d in Black individuals were 1.32 (95% CI, 1.07-1.63) for total CVD, 1.48 (95% CI, 1.05-2.09) for CHD, and 1.27 (95% CI, 1.04-1.56) for other diseases; for White individuals, HRs were 1.48 (95% CI, 1.04-2.10) for total CVD and 10.99 (95% CI, 3.04-39.76) for heart failure. In Black individuals, after adjustment for potential confounders, a 1000-mg increase in daily sodium intake was associated with a 3% higher risk of death from all-cause, 7% higher risk from total CVD (HR, 1.07; 95% CI, 1.03-1.10), 8% higher risk from CHD (HR, 1.08; 95% CI, 1.02-1.14). In White individuals, a 1000-mg increase in daily sodium intake was associated with an 8% higher risk from total CVD (HR, 1.08; 95% CI, 1.02-1.14), 13% higher risk from CHD (HR, 1.13; 95% CI, 1.03-1.23), and 55% higher risk of death from heart failure. Significant interactions between sodium intake and race were found for all-cause (*P* for interaction <.001), total CVD (*P* for interaction = .01), and heart failure mortality (*P* for interaction = .03).

**Table 2.** All-Cause and Cause-Specific Mortality in Relation to Dietary Sodium Intake

Causes of death	No. of participants (No. of deaths)	Hazard ratio (95% CI) by dietary sodium intake, mg/d^a^					1000-mg/d increment	*P* for interaction^b^
		<2300	2300-3450	3451-4600	4601-6900	>6900		
All-cause								
Black Americans	46 185 (12 256)	1 [Reference]	1.02 (0.96-1.08)	1.08 (1.01-1.15)	1.10 (1.01-1.19)	1.21 (1.07-1.36)	1.03 (1.01-1.05)	<.001
White Americans	18 144 (5555)	1 [Reference]	1.06 (0.97-1.15)	0.98 (0.89-1.09)	1.13 (1.00-1.27)	1.09 (0.91-1.32)	1.01 (0.98-1.04)	
Total CVD								
Black Americans	46 185 (4112)	1 [Reference]	1.00 (0.90-1.10)	1.10 (0.98-1.23)	1.11 (0.96-1.27)	1.32 (1.07-1.63)	1.07 (1.03-1.10)	.01
White Americans	18 144 (1589)	1 [Reference]	1.09 (0.94-1.27)	1.03 (0.85-1.24)	1.18 (0.94-1.48)	1.48 (1.04-2.10)	1.08 (1.02-1.14)	
CHD								
Black Americans	46 185 (1480)	1 [Reference]	0.96 (0.81-1.14)	1.21 (1.00-1.46)	1.21 (0.96-1.53)	1.48 (1.05-2.09)	1.08 (1.02-1.14)	.33
White Americans	18 144 (736)	1 [Reference]	1.05 (0.83-1.32)	1.04 (0.79-1.37)	1.16 (0.83-1.62)	1.35 (0.80-2.28)	1.13 (1.03-1.23)	
Stroke								
Black Americans	46 185 (701)	1 [Reference]	0.95 (0.75-1.21)	0.88 (0.66-1.16)	0.99 (0.71-1.37)	1.10 (0.67-1.80)	1.11 (1.03-1.20)	.39
White Americans	18 144 (204)	1 [Reference]	1.12 (0.73-1.72)	1.13 (0.66-1.92)	1.64 (0.86-3.16)	1.17 (0.39-3.54)	1.02 (0.86-1.21)	
Heart failure								
Black Americans	46 185 (354)	1 [Reference]	1.15 (0.84-1.59)	1.07 (0.72-1.59)	1.06 (0.65-1.74)	1.29 (0.60-2.79)	1.11 (0.98-1.26)	.03
White Americans	18 144 (116)	1 [Reference]	1.00 (0.56-1.77)	1.24 (0.60-2.54)	2.50 (1.05-5.93)	10.99 (3.04-39.76)	1.55 (1.20-2.01)	
Cancer								
Black Americans	46 185 (2878)	1 [Reference]	0.92 (0.82-1.04)	1.02 (0.89-1.16)	1.04 (0.89-1.22)	1.05 (0.82-1.33)	1.00 (0.96-1.03)	.18
White Americans	18 144 (1154)	1 [Reference]	1.16 (0.97-1.39)	1.19 (0.96-1.49)	1.39 (1.06-1.81)	1.30 (0.85-1.98)	0.99 (0.93-1.06)	
Other diseases								
Black Americans	46 185 (4444)	1 [Reference]	1.13 (1.03-1.25)	1.12 (1.00-1.25)	1.17 (1.03-1.34)	1.27 (1.04-1.56)	1.04 (1.01-1.07)	.32
White Americans	18 144 (2263)	1 [Reference]	1.03 (0.91-1.17)	0.96 (0.82-1.12)	1.02 (0.85-1.24)	0.81 (0.60-1.10)	0.97 (0.92-1.01)	

Abbreviations: CHD, coronary heart disease; CVD, cardiovascular disease.

^a^ Adjusted for age, sex, education, income, marital status, medical insurance, smoking status, physical activity, alcohol consumption, body mass index, total energy intake, and healthy eating index.

^b^ Tested by the likelihood ratio test, comparing models with and without the multiplicative interaction term of sodium intake (continuous) × race.

**Figure 1.**
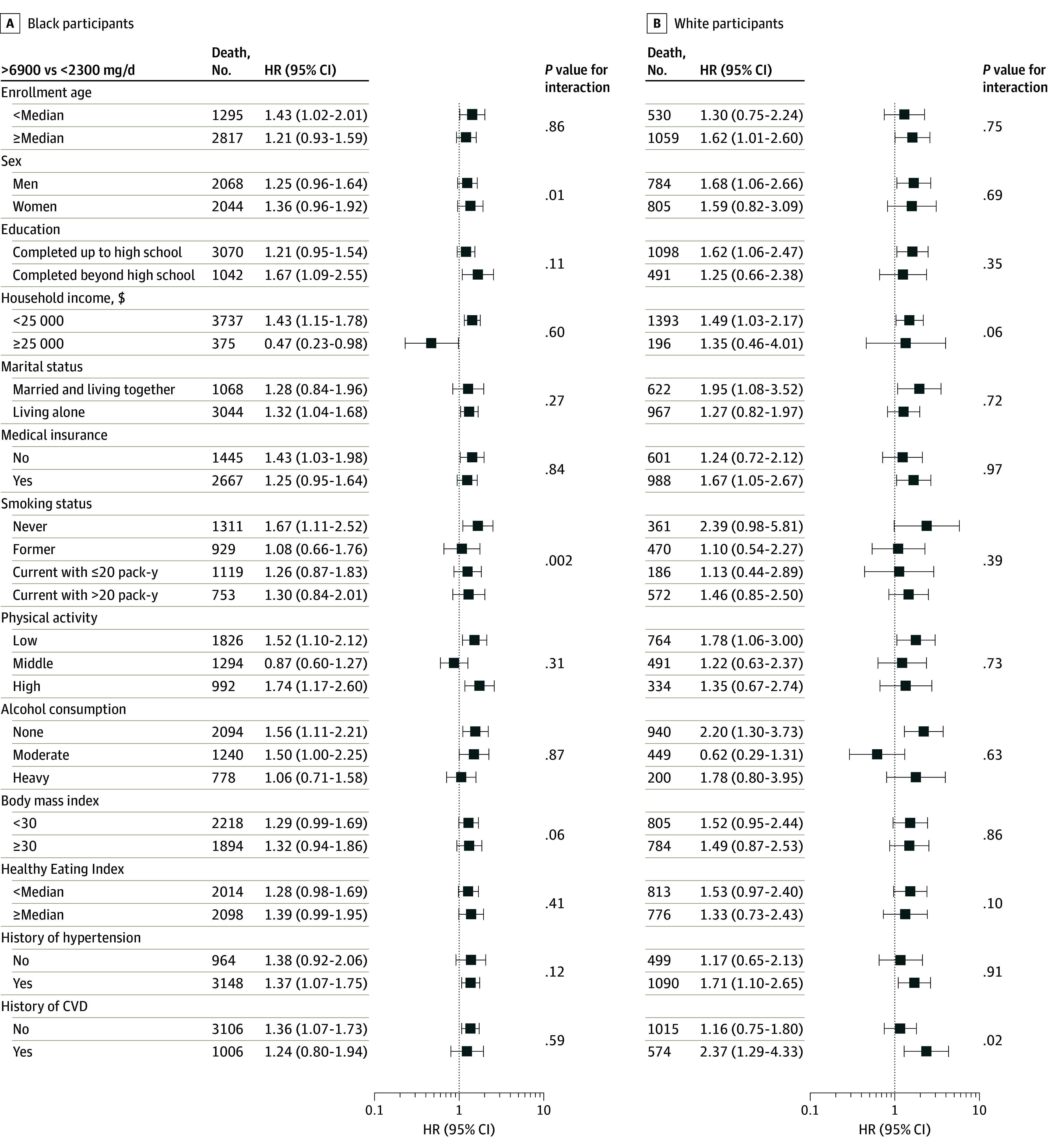
Total Cardiovascular Disease Mortality in Relation to Dietary Sodium Intake: Subgroup Analysis by Baseline Characteristics Hazard ratios (HRs) and 95% CIs for more than 6900 mg/d vs less than 2300 mg/d were estimated after adjustment for age, sex, education, income, marital status, medical insurance, smoking, physical activity, alcohol consumption, body mass index, total energy intake, and healthy eating index. Living alone included individuals who never married and those who were separated, divorced, widowed, or single. Physical activity was categorized by tertiles of the total metabolic equivalent of task hours per week. Alcohol consumption was defined as nondrinkers, 0 g/d; moderate drinkers, more than 0 but 28 or less g/d for men or more than 0 but 14 or less g/d for women; and heavy drinkers, more than 28 g/d for men or more than 14 g/d for women. Interaction was tested by the likelihood ratio test, comparing models with and without the multiplicative interaction term of sodium intake (continuous) × each stratum variable. Error bars represent the 95% CIs.

**Figure 2.**
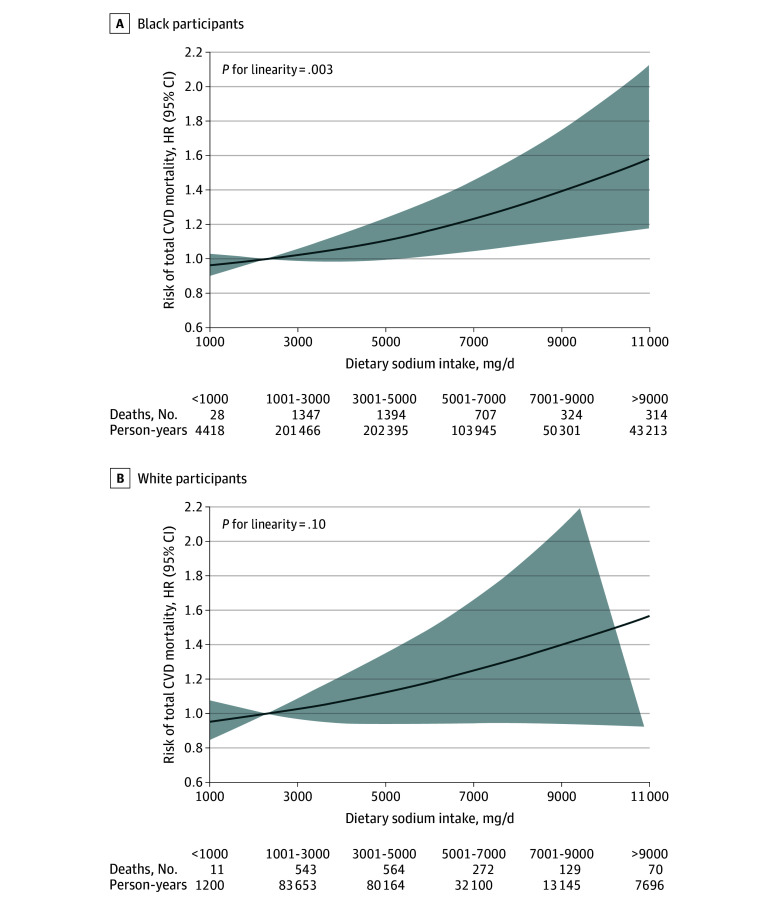
Dose-Response Association of Dietary Sodium Intake With Total Cardiovascular Disease Mortality Solid lines represent the hazard ratios and shaded areas represent the 95% CIs. The reference was set at 2300 mg/d, with 3 knots fitted at the 5th, 50th, and 95th percentiles. Participants with the highest 1% of sodium intake were excluded to reduce the noise of extreme outliers. All models were adjusted for age, sex, enrollment source, education, income, marital status, medical insurance, smoking, physical activity, alcohol consumption, body mass index, total energy intake, and healthy eating index.

**Figure 3.**
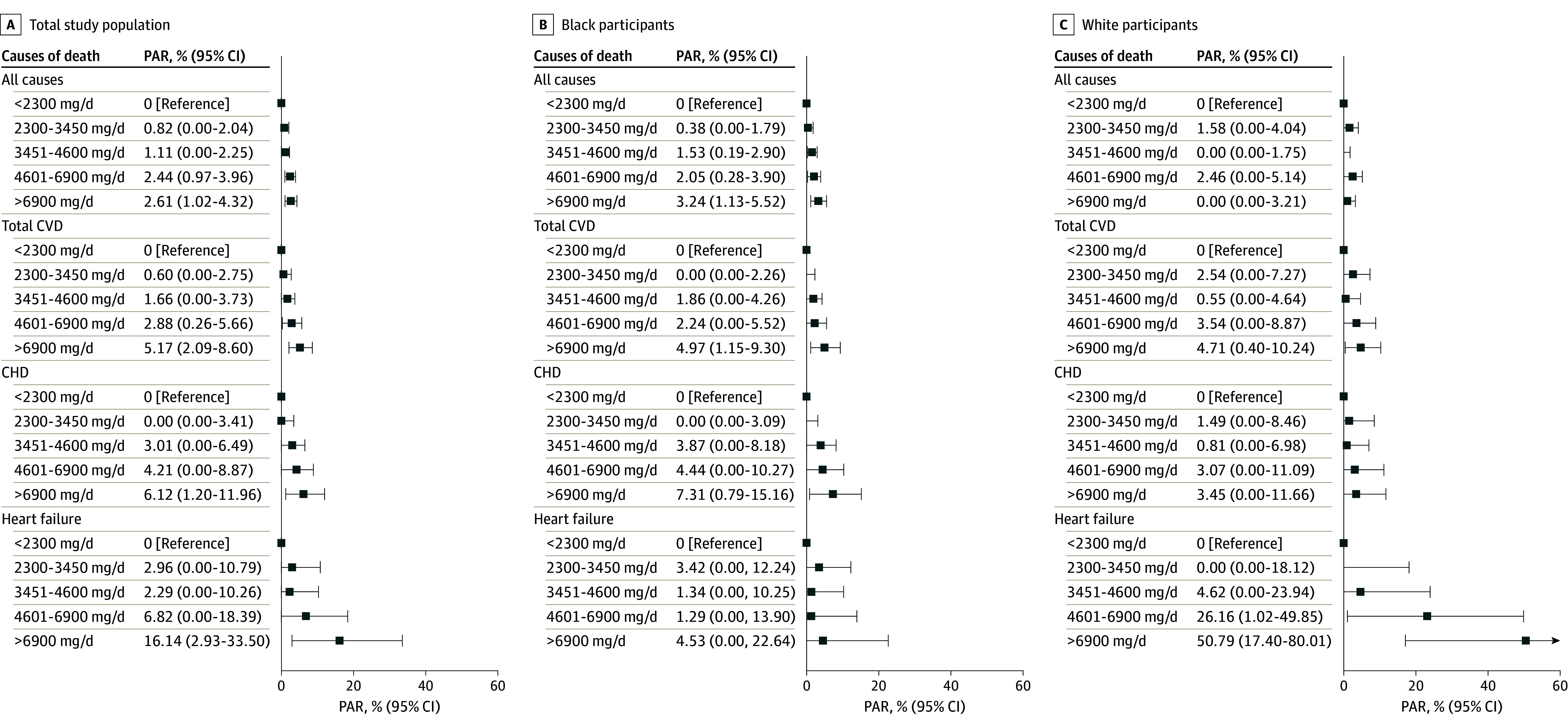
Population-Attributable Risk (PAR) for Cause-Specific Mortality by Dietary Sodium Intake Among Low-Income Black and White Americans The calculated PARs represent a value of 0 if they were negative for mortality outcomes. Error bars represent the 95% CIs for the population-attributable risk estimates. CHD indicates coronary heart disease; CVD, cardiovascular disease; PAR, population-attributable risk.

In stratified analyses (Figure 1), overall association patterns between excess sodium intake and total CVD mortality in Black individuals appeared to be more evident among never-smokers than among ever-smokers (*P* for interaction = .002): multivariable-adjusted HRs for more than 6900 mg/d vs less than 2300 mg/d were 1.67 (95% CI, 1.11-2.52) for never smokers, 1.08 (95% CI, 0.66-1.76) for former smokers, 1.26 (95% CI, 0.87-1.83) for current smokers with 20 or fewer pack-years, and 1.30 (95% CI, 0.84-2.01) for current smokers with more than 20 pack-years. Meanwhile, White individuals with a history of CVD showed over 2-fold increased CVD mortality in relation to sodium intake more than 6900 mg/d, but no association was observed in their CVD-free counterparts: HRs for more than 6900 mg/d vs less than 2300 mg/d, 2.37 (95% CI, 1.29-4.33) and 1.16 (95% CI, 0.75-1.80), respectively (*P* for interaction = .02). Restricted cubic spline analyses suggested that the association between dietary sodium intake and total CVD mortality follows a near linear dose-response pattern in both Black and White individuals (Figure 2). No significant dose-response associations were observed for any other mortality outcomes. The overall PAR associated with exceeding 2300 mg of dietary sodium per day was approximately 10% for total CVD mortality, 13% for CHD mortality, and about 30% for heart failure mortality in our underserved population (Figure 3). The sodium-mortality associations were not changed in sensitivity analyses, indicating the robustness of our findings (eTable 5, eTable 6, eTable 7, eTable 8, and eTable 9 in Supplement 1).

## Discussion

In this cohort study of low-income Black and White Americans, we found approximately 80% of study participants exceeded current guidelines for sodium intake of 2300 mg or less per day. The average daily intake was 4512 mg in Black Americans and 4041 mg in White Americans—much higher than the average estimate of 3400 mg/d among the general US adult population.^4^ Excessive sodium intake was associated with increased hazards of mortality, mainly due to CVD-related deaths, after adjusting for potential confounders, including behavior factors and dietary quality. PAR analyses suggest that excessive dietary sodium may be responsible for approximately 10% of total CVD deaths and about 30% of heart failure deaths in this low-income population.

High sodium intake can promote oxidative stress, endothelial dysfunction, and increased arterial stiffness^22^ and further lead to an imbalance in immune homeostasis and a persistent proinflammatory state,^23,24,25,26^ all of which are significant predictors of CVD and hypertension. The Dietary Approaches to Stop Hypertension (DASH) diet sodium trial demonstrated blood pressure decreases with reduction in sodium intake,^27^ as well as improvements in vascular endothelial function.^28^ Given the biological connection between high sodium intake and blood pressure in left-ventricular hypertrophy,^29^ it was hypothesized that the harmful effects of excess sodium would be further intensified among hypertensive individuals, particularly Black people who have significantly higher rates of hypertension than other populations.^30^ However, in the current study, we found no evidence of effect modification by hypertension in the sodium-CVD mortality association. Furthermore, additional adjustments for history of CVD and hypertension did not change the primary associations. These findings suggest that high sodium diets may confer detrimental health consequences beyond their effects on hypertension.

The role of dietary sodium in increasing the risk of death, particularly from CVD, has been consistently reported. A meta-analysis of 23 cohort studies and 2 follow-up studies of randomized clinical trials reported a 16% increase in all-cause mortality associated with high sodium intake and a 9% risk reduction associated with low sodium intake.^11^ The third NHANES study showed 20% higher all-cause mortality per 1000-mg increase in daily sodium intake.^9^ Also, diets high in sodium were associated with an increased risk of congestive heart failure among overweight Americans^31^ and an increased risk of death among patients diagnosed with ambulatory heart failure.^32^ Meanwhile, a 10-year follow-up study of 2642 elderly Americans found nonsignificant positive associations between consuming more than 2300 mg/d of sodium and all-cause mortality, incident CVD, or incident heart failure.^12^ Another systematic review and meta-analysis showed that sodium intake was positively associated with deaths from CHD (RR, 1.32; 95% CI, 1.13-1.53) and stroke (RR, 1.63; 95% CI, 1.27-2.10), but null associations were found for all-cause mortality or incident CVD and CHD.^33^ It is worth noting that low-SES individuals and Black adults are disproportionately exposed to low-quality diets and have a higher risk of premature death than others^14,34^; however, they are relatively underrepresented in epidemiologic studies on this matter to date. Findings from the current study of predominantly low-income Black and White Americans not only demonstrated their substantially higher exposure to excessive sodium intake, but provided compelling evidence of the association between excessive sodium and CVD-related deaths in this underserved population.

Large amounts of dietary sodium intake from processed/restaurant foods have been documented in the US for several decades.^35^ Also, snacking raises sodium overconsumption dramatically, especially among individuals with the lowest income, uneducated people, and Black individuals.^36^ In the same vein, our findings affirmed that (1) Black Americans consumed more sodium than White Americans with the same SES; and (2) individuals with lower SES appeared to eat an even greater quantity of sodium overall, regardless of race. It is noteworthy that low-income Black Americans consumed almost double the recommended amount of sodium (4512 mg/d vs 2300 mg/d). The average sodium intake in our low-income cohort exceeded that of general US adults (3400 mg/d)^4^ by 19% for White individuals and 33% for Black individuals. It is imperative to emphasize that low-income communities exhibit a higher concentration of fast-food restaurants and convenience stores with limited healthy food options, particularly in areas where racial and ethnic minorities are overrepresented^37,38^; this contributes to higher sodium exposure in these underserved groups. Economic barriers and unfavorable nutrition environments, including food insecurity and a lack of access to nutritious foods, are significant determinants of poor diet quality, including excessive sodium intake.^37^ Providing accessible healthy diet resources and increasing knowledge on the potential harms associated with high sodium intake would be the first steps in reducing racial, ethnic, and socioeconomic disparities in everyday diets and health outcomes.

Our PAR findings suggest that excessive sodium was likely responsible for about 10% to 30% of CVD-related deaths among underserved Americans. Although causality cannot be inferred from a single observational study like ours, a study using US nationally representative data reported that high sodium accounted for the largest number of estimated diet-related cardiometabolic deaths in 2012 (9.5% of all cardiometabolic deaths),^10^ particularly deaths from CHD (10.2% of CHD deaths). These findings highlight that dietary sodium reduction can be one of the most effective strategies for improving cardiovascular health. Of note, our study found a significant effect modification by race for the sodium–CVD mortality association, with higher risk estimates seen among low-income White individuals than their Black counterparts. This heterogeneity might be attributable to the complex and multidimensional nature of racial diversity. Race is a sociological construct with the potential to influence physiological mechanisms that regulate health-influencing factors through certain variations in dietary composition, lifestyle habits, and levels of psychosocial stress experienced by Black and White individuals.^39^ Indeed, the baseline characteristics of our study participants significantly differed across races, potentially accounting for the observed racial difference in the sodium-CVD mortality association. Given racial disparities in CVD-related disorders linked to race-specific daily dietary patterns,^40^ it is possible that differences in nutrient composition (eg, potassium, fiber, and so forth) between Black and White people may impact the sodium-CVD mortality association. Further investigation focusing on social determinants of nutrition beyond sodium is necessary to tackle racial, ethnic, and socioeconomic disparities in health outcomes.

This prospective investigation enabled us to address associations of dietary sodium intake with mortality outcomes among low-income Black and White Americans, who are disproportionately exposed to excessive sodium intake. Our large sample size, long-term follow-up, and a wide range of detailed baseline information allowed for in-depth analyses in consideration of subgroup differences and comprehensive adjustments for CVD risk factors and confounders, as well as evaluations of the mortality burdens attributable to excessive sodium intake among underserved Americans. As our study participants have similar SES backgrounds and comparable access to medical care, we had the unique opportunity to evaluate potential racial differences in sodium and mortality associations with minimum confounding from SES.

### Limitations

Study limitations should be noted. First, we could not measure sodium intake using objective methods, such as 24-hour urine^13,41^ or blood samples.^25^ Because we estimated dietary sodium intake based on FFQ data only, measurement errors in estimating sodium intake might exist.^42^ Such exposure misclassification is likely to be nondifferential given our prospective study design, leading to an underestimated risk. Second, sodium intake was assessed only at baseline; thus, dietary changes over time were not captured. Third, despite the important interplay between sodium and potassium in controlling CVD-related conditions,^39^ we could not address the impact of potassium intake on the sodium-mortality association, which calls for future comprehensive investigations. Fourth, despite our extensive adjustments for established CVD risk factors and potential confounders, we cannot exclude the possibility of residual confounding. Additionally, the treatment effects of comorbid conditions could not be controlled due to a lack of information. Fifth, the possible misclassification of underlying causes of death is another concern. Specific causes of death are often imprecisely captured in the death registry, and some reported causes, such as heart failure, could reflect multiple underlying causes. Additionally, small sample sizes included in some subgroup analyses, eg, heart failure mortality, might result in risk estimates that were not statistically significant or less reliable.

## Conclusions

In this cohort study of low-income Black and White Americans, we found that over 80% of this underserved population did not adhere to the current dietary guidelines for sodium intake, despite substantial public health efforts to lower sodium intake in Americans during recent decades. High sodium intake appeared to account for about 10% to 30% of CVD-related deaths among them. Developing effective dietary modification strategies tailored to this marginalized population is urgently needed to promote health and prevent ever-increasing health disparities in the US.

## Notes

Supplement 1. **eTable 1. **Dietary Sodium Intakes by Educational Attainment and Annual Household Income **eTable 2. **Baseline Characteristics of Study Participants by Race **eTable 3. **Baseline Characteristics of Study Participants: Black Americans vs White Americans **eTable 4. **Race-Specific Deaths Across Dietary Sodium Intake **eTable 5. **All-Cause and Cause-Specific Mortality in Relation to Dietary Sodium Intake: Further Adjusting for History of Hypertension and Cardiovascular Disease **eTable 6. **All-Cause and Cause-Specific Mortality in Relation to Dietary Sodium Intake: Excluding First 2 Years of Follow-up **eTable 7. **All-Cause and Cause-Specific Mortality in Relation to Dietary Sodium Intake: Excluding Individuals With History of Cardiovascular Disease **eTable 8. **All-Cause and Cause-Specific Mortality in Relation to Dietary Sodium Intake: Further Adjusting for Comorbidity Index **eTable 9. **All-Cause and Cause-Specific Mortality in Relation to Dietary Sodium Intake: Competing Risk Analysis **eFigure. **Conceptual Framework for Research Questions

Supplement 2.
Data Sharing Statement
