# Supplementary material for: Sodium Intake and Cause-Specific Mortality Among Predominantly Low-Income Black and White US Residents
Source: JAMA Netw Open. 2024 Mar 26;7(3):e243802. doi: 10.1001/jamanetworkopen.2024.3802 (PMC10966417; doi:10.1001/jamanetworkopen.2024.3802)
Supplement: Supplement 1. — eTable 1. Dietary Sodium Intakes by Educational Attainment and Annual Household Income eTable 2. Baseline Characteristics of Study Participants by Race eTable 3. Baseline Characteristics of Study Participants: Black Americans vs White Americans eTable 4. Race-Specific Deaths Across Dietary Sodium Intake eTable 5. All-Cause and Cause-Specific Mortality in Relation to Dietary Sodium Intake: Further Adjusting for History of Hypertension and Cardiovascular Disease eTable 6. All-Cause and Cause-Specific Mortality in Relation to Dietary Sodium Intake: Excluding First 2 Years of Follow-up eTable 7. All-Cause and Cause-Specific Mortality in Relation to Dietary Sodium Intake: Excluding Individuals With History of Cardiovascular Disease eTable 8. All-Cause and Cause-Specific Mortality in Relation to Dietary Sodium Intake: Further Adjusting for Comorbidity Index eTable 9. All-Cause and Cause-Specific Mortality in Relation to Dietary Sodium Intake: Competing Risk Analysis eFigure. Conceptual Framework for Research Questions [file jamanetwopen-e243802-s001.pdf]

Supplemental Online Content

Yoon HS, Cai Q, Yang JJ, et al. Sodium intake and cause-specific mortality among predominantly low-income Black and White Americans. *JAMA Netw Open*. 2024;7(3):e243802. doi:10.1001/jamanetworkopen.2024.3802

eTable 1. Dietary Sodium Intakes by Educational Attainment and Annual Household Income

eTable 2. Baseline Characteristics of Study Participants by Race

eTable 3. Baseline Characteristics of Study Participants: Black Americans vs White Americans

eTable 4. Race-Specific Deaths Across Dietary Sodium Intake

eTable 5. All-Cause and Cause-Specific Mortality in Relation to Dietary Sodium Intake: Further Adjusting for History of Hypertension and Cardiovascular Disease

eTable 6. All-Cause and Cause-Specific Mortality in Relation to Dietary Sodium Intake: Excluding First 2 Years of Follow-up

eTable 7. All-Cause and Cause-Specific Mortality in Relation to Dietary Sodium Intake: Excluding Individuals With History of Cardiovascular Disease

eTable 8. All-Cause and Cause-Specific Mortality in Relation to Dietary Sodium Intake: Further Adjusting for Comorbidity Index

eTable 9. All-Cause and Cause-Specific Mortality in Relation to Dietary Sodium Intake: Competing Risk Analysis

eFigure. Conceptual Framework for Research Questions

This supplemental material has been provided by the authors to give readers additional information about their work.

© 2024 Yoon HS et al. *JAMA Network Open.*

eTable 1. Dietary Sodium Intakes by Educational Attainment and Annual Household Income

Baseline Characteristics	Black Americans, *N*=46,185		White Americans, *N*=18,144	
	N (%)	Sodium Intake (mg/day), Mean±SD	N (%)	Sodium Intake (mg/day), Mean±SD
**Total Participants**	46,185 (100)	4,512 ± 2,632	18,144 (100)	4,041 ± 2,227
**Education**				
Less than 9 years of schooling	3,905 (8)	4,273 ± 2,500	1,730 (10)	4,062 ± 2,259
High school dropouts	11,273 (24)	4,653 ± 2,757	3,468 (19)	4,184 ± 2,366
Completed high school	16,158 (35)	4,603 ± 2,703	6,420 (35)	4,157 ± 2,339
Vocational or technical training	2,369 (5)	4,417 ± 2,514	899 (5)	3,835 ± 2,066
Some college or junior college	8,678 (19)	4,476 ± 2,505	3,553 (20)	3,939 ± 2,092
Graduated from college	2,732 (6)	4,206 ± 2,433	1,364 (7)	3,788 ± 1,886
Graduate school: master's degree	806 (2)	3,801 ± 2,047	532(3)	3,499 ± 1,653
Beyond a master's degree [a]	264 (<1)	3,759 ± 2,310	178 (1)	3,563 ± 1,542
**Annual household Income**				
< $15,000	28,872 (63)	4,593 ± 2,695	10,301 (57)	4,145 ± 2,314
≥ $15,000 to < $25,000	10,392 (22)	4,486 ± 2,594	3,728 (20)	4,129 ± 2,266
≥ $25,000 to < $50,000	5,235 (11)	4,278 ± 2,409	2,524 (14)	3,848 ± 2,058
≥ $50,000	1,686 (4)	3,999 ± 2,314	1,591 (9)	3,471 ± 1,627

Abbreviation: mg/day, milligrams per day; n, number; SD, standard deviation.

a. Included doctors, dentists, lawyers, and individuals with PhD's degree

© 2024 Yoon H-S et al. *JAMA Network Open.*

eTable 2. Baseline Characteristics of Study Participants by Race

Baseline Characteristics	Black Americans, *N*=46,185				White Americans, *N*=18,144			
	Dietary Sodium Intake (mg/day),[a] N (%)				Dietary Sodium Intake (mg/day),[a] N (%)			
	< 2,300 (N=8,703)	3,451-4,600 (N=8,943)	> 6,900 (N=7,549)	*p*	< 2,300 (N=3,713)	3,451-4,600 (N=3,708)	> 6,900 (N=1,875)	*p*
**Enrollment age,** mean±SD years	53.0±9.0	51.4±8.5	48.6±7.0	< 0.001	55.4±9.5	53.0±9.1	49.7±7.8	<0.001
**Sex**								
Men	1,979 (23)	3,597 (40)	4,747 (63)	< 0.001	517 (14)	1,315 (35)	1,314 (70)	<0.001
Women	6,724 (77)	5,346 (60)	2,802 (37)		3,196 (86)	2,393 (65)	561 (30)	
**Education**								
≤ Completed high school	5,874 (67)	6,027 (67)	5,382 (71)	< 0.001	2,377 (64)	2,330 (63)	1,367 (73)	<0.001
> Completed high school	2,829 (33)	2,916 (33)	2,167 (29)		1,336 (36)	1,378 (37)	508 (27)	
**Annual household Income**								
< $15,000	2,879 (33)	2,914 (33)	2,573 (34)	< 0.001	2,085 (56)	2,078 (56)	1,199 (64)	<0.001
≥ $15,000 to < $25,000	2,995 (35)	3,113 (35)	2,809 (37)		724 (20)	767 (21)	406 (22)	
≥ $25,000 to < $50,000	2,018 (23)	2,179 (24)	1,717 (23)		536 (14)	542 (14)	203 (11)	
≥ $50,000	811 (9)	737 (8)	450 (6)		368 (10)	321 (9)	67 (3)	
**Marital status**								
Married/Living with a partner	2,371 (27)	2,665 (30)	1,817 (24)	< 0.001	1,556 (42)	1,656 (45)	649 (35)	<0.001
Living alone [b]	6,332 (73)	6,278 (70)	5,732 (76)		2,157 (58)	2,052 (55)	1,226 (65)	
**Having medical insurance**	5,272 (61)	5,214 (58)	3,772 (50)	< 0.001	2,362 (64)	2,112 (57)	808 (43)	<0.001
**Smoking status**								
Never	3,997 (46)	3,344 (37)	1,953 (26)	< 0.001	1,368 (37)	1,127 (30)	362 (19)	<0.001
Former	1,924 (22)	1,800 (20)	1,077 (14)		1,058 (28)	1,008 (27)	353 (19)	
Current with ≤ 20 pack-years	1,922 (22)	2,483 (28)	3,006 (40)		429 (12)	470 (13)	352 (19)	
Current with > 20 pack-years	860 (10)	1,316 (15)	1,513 (20)		858 (23)	1,103 (30)	808 (43)	
**Physical activity** [c]								
Low	3,442 (40)	2,917 (33)	1,916 (25)	< 0.001	1,513 (41)	1,304 (35)	531 (28)	<0.001
Middle	2,894 (33)	3,041 (34)	2,332 (31)		1,168 (31)	1,220 (33)	539 (29)	
High	2,367 (27)	2,985 (33)	3,301 (44)		1,032 (28)	1,184 (32)	805 (43)	
**Alcohol consumption** [d]								
None	4,944 (57)	4,094 (46)	2,453 (33)	< 0.001	2,313 (62)	1,962 (53)	803 (43)	<0.001
Moderate	2,841 (33)	3,209 (36)	2,671 (35)		1,155 (31)	1,330 (36)	625 (33)	
Heavy	918 (10)	1,640 (18)	2,425 (32)		245 (7)	416 (11)	447 (24)	

© 2024 Yoon H-S et al. *JAMA Network Open.*

**Body mass index**								
< 30 kg/m^2	3,901 (45)	4,718 (53)	4,939 (65)	< 0.001	2,022 (54)	1,991 (54)	1,168 (62)	<0.001
≥ 30 kg/m^2	4,802 (55)	4,225 (47)	2,610 (35)		1,691 (46)	1,717 (64)	707 (38)	
**Healthy Eating Index,** mean±SD	60.8±12.7	56.8±11.6	54.7±9.2	< 0.001	59.7±13.6	56.0±12.1	51.8±9.3	<0.001
**Disease history**								
Hypertension	5,692 (65)	5,225 (58)	3,542 (47)	< 0.001	1,968 (53)	1,926 (52)	821 (44)	<0.001
CVD	1,035 (12)	942 (10)	711 (9)	< 0.001	566 (15)	556 (15)	303 (16)	0.62
**Comorbidity index,** mean±SD	2.0±1.4	1.8±1.5	1.6±1.5	< 0.001	2.3±1.6	2.2±1.6	2.0±1.6	<0.001
**Cause of death,** *n*								
All causes	2,219	2,381	2,076		1,045	1,075	673	
Total CVD	790	808	671		299	309	205	
Cancer	529	560	487		207	236	127	
Other diseases	785	848	721		448	451	235	

Abbreviation: mg/day, milligrams per day; n, number; SD, standard deviation; CVD, cardiovascular disease.

a. Mean (SD) intake was 4,512 (2,632) mg/day among Black individuals and 4,042 (2,227) mg/day among White individuals

b. Included individuals who never married and those who were separated, divorced, widowed, or single

c. Defined by tertiles of the total metabolic equivalent of task hours per week

d. Defined as nondrinkers, 0 gram/day; moderate drinkers, >0 but ≤28 grams/day for men or >0 but ≤14 grams/day for women; and heavy drinkers, >28 grams/day for men or >14 grams/day for women

© 2024 Yoon H-S et al. *JAMA Network Open.*

eTable 3. Baseline Characteristics of Study Participants: Black Americans vs White Americans

Baseline Characteristics	Black Americans, N (%) (*N*=46,185)	White Americans, N (%) (*N*=18,144)	*p*
**Enrollment age,** mean±SD years	51.3±8.6	53.3±9.3	<0.001
**Sex**			
Men	18,885 (41)	6,289 (35)	<0.001
Women	27,300 (59)	11,855 (65)	
**Education**			
≤ Completed high school	31,336 (68)	11,618 (64)	<0.001
> Completed high school	14,849 (32)	6,526 (36)	
**Annual household Income**			
< $15,000	28,872 (63)	10,301 (57)	<0.001
≥ $15,000 to < $25,000	10,392 (22)	3,728 (20)	
≥ $25,000 to < $50,000	5,235 (11)	2,524 (14)	
≥ $50,000	1,686 (4)	1,591 (9)	
**Marital status**			
Married/Living with a partner	12,909 (28)	7,798 (43)	<0.001
Living alone [a]	33,276 (72)	10,346 (57)	
**Having medical insurance**	26,364 (57)	10,301 (57)	0.48
**Smoking status**			
Never	16,999 (37)	5,572 (31)	< 0.001
Former	9,013 (19)	4,691 (26)	
Current with ≤ 20 pack-years	13,363 (29)	2,388 (13)	
Current with > 20 pack-years	6,810 (15)	5,493 (30)	
**Physical activity [b]**			
Low	15,218 (33)	6,513 (36)	<0.001
Middle	15,227 (33)	5,782 (32)	
High	15,740 (34)	5,849 (32)	
**Alcohol consumption [c]**			
None	21,067 (46)	9,723 (53)	<0.001
Moderate	16,063 (35)	6,311 (35)	
Heavy	9,055 (19)	2,110 (12)	
**Body mass index (kg/m^2),** mean±SD	30.5±7.6	30.3±7.7	<0.001
**BMI status**			
< 18.5 kg/m^2	537 (1)	286 (2)	<0.001

© 2024 Yoon H-S et al. *JAMA Network Open.*

≥ 18.5 to < 25.0	10,846 (23)	4,480 (25)	
≥ 25.0 to < 30.0	13,422 (29)	5,215 (29)	
≥ 30.0 to < 35.0	10,427 (23)	4,088 (22)	
≥ 30.0 kg/m^2	10,953 (24)	4,075 (22)	
**Healthy Eating Index,** mean±SD	57.5±11.6	56.5±12.4	<0.001
**Total Energy Intake,** mean±SD	2,704±1,529	2,310±1,269	<0.001
**Sodium Intake (mg/day),** mean±SD	4,512±2,632	4,042±2,227	<0.001
< 2,300	8,703 (19)	3,713 (21)	<0.001
2,300-3,450	10,917 (24)	5,192 (29)	
3,451-4,600	8,943 (19)	3,708 (20)	
4,601-6,900	10,073 (22)	3,656 (20)	
> 6,900	7,549 (16)	1,875 (10)	
**Disease history**			
Hypertension	26,754 (58)	9,282 (51)	<0.001
Diabetes	10,352 (22)	3,802 (21)	<0.001
Dyslipidemia	13,487 (29)	7,452 (41)	<0.001
CVD	5,007 (11)	2,823 (15)	<0.001
**Comorbidity index,** mean±SD	1.8±1.5	2.2±1.6	<0.001
**Cause of death,** *n*			
All causes	12,256	5,555	
Total CVD	4,112	1,589	
Cancer	2,878	1,154	
Other diseases	4,444	2,263	

Abbreviation: mg/day, milligrams per day; n, number; SD, standard deviation; CVD, cardiovascular disease.

a. Included individuals who never married and those who were separated, divorced, widowed, or single

b. Defined by tertiles of the total metabolic equivalent of task hours per week

c. Defined as nondrinkers, 0 gram/day; moderate drinkers, >0 but ≤28 grams/day for men or >0 but ≤14 grams/day for women; and heavy drinkers, >28 grams/day for men or >14 grams/day for women

© 2024 Yoon H-S et al. *JAMA Network Open.*

eTable 4. Race-Specific Deaths Across Dietary Sodium Intake

Causes of death	No. of participants (no. of deaths)	Deaths by Dietary Sodium Intake (mg/day), N (%)				
		< 2,300	2,300-3,450	3,451-4,600	4,601-6,900	> 6,900
**All-cause**						
Black Americans	46,185 (12,256)	2,219 (26)	2,827 (26)	2,381 (27)	2,753 (27)	2,076 (28)
White Americans	18,144 (5,555)	1,045 (28)	1,504 (29)	1,075 (29)	1,258 (34)	673 (36)
**Total CVD**						
Black Americans	46,185 (4,112)	790 (9)	959 (9)	808 (9)	884 (9)	671 (9)
White Americans	18,144 (1,589)	299 (8)	433 (8)	309 (8)	343 (9)	205 (11)
**CHD**						
Black Americans	46,185 (1,480)	272 (3)	318 (3)	306 (3)	334 (3)	250 (3)
White Americans	18,144 (736)	139 (4)	196 (4)	149 (4)	162 (4)	90 (5)
**Stroke**						
Black Americans	46,185 (701)	137 (2)	166 (2)	121 (1)	155 (2)	122 (2)
White Americans	18,144 (204)	40 (1)	57 (1)	40 (1)	52 (1)	15 (1)
**Heart failure**						
Black Americans	46,185 (354)	74 (1)	100 (1)	66 (1)	67 (1)	47 (1)
White Americans	18,144 (116)	26 (1)	26 (1)	18 (1)	23 (1)	23 (1)
**Cancer**						
Black Americans	46,185 (2,878)	529 (6)	625 (6)	560 (6)	677 (7)	487 (7)
White Americans	18,144 (1,154)	207 (6)	317 (6)	236 (6)	267 (7)	127 (7)
**Other diseases**						
Black Americans	46,185 (4,444)	785 (9)	1,100 (10)	848 (10)	990 (10)	721 (10)
White Americans	18,144 (2,263)	448 (12)	630 (12)	451 (12)	499 (14)	235 (13)

Abbreviation: mg/day, milligrams per day; no, number; CVD, cardiovascular disease; CHD, coronary heart disease; ref, reference.

© 2024 Yoon H-S et al. *JAMA Network Open.*

eTable 5. All-Cause and Cause-Specific Mortality in Relation to Dietary Sodium Intake: Further Adjusting for History of Hypertension and Cardiovascular Disease

Causes of death	No. of participants (no. of deaths)	Hazard Ratio (95% CI) by Dietary Sodium Intake (mg/day) [a]					1,000 mg/day Increment	*P* Interaction [b]
		< 2,300	2,300-3,450	3,451-4,600	4,601-6,900	> 6,900		
**All-cause**								
Black Americans	46,185 (12,256)	1.00 (Ref.)	1.02 (0.96-1.08)	1.09 (1.02-1.17)	1.12 (1.03-1.21)	1.24 (1.10-1.40)	1.04 (1.02-1.06)	0.003
White Americans	18,144 (5,555)	1.00 (Ref.)	1.05 (0.97-1.15)	0.99 (0.90-1.09)	1.12 (0.99-1.26)	1.10 (0.92-1.33)	1.01 (0.98-1.04)	
**Total CVD**								
Black Americans	46,185 (4,112)	1.00 (Ref.)	1.00 (0.90-1.10)	1.12 (1.00-1.26)	1.14 (0.99-1.31)	1.39 (1.13-1.71)	1.07 (1.04-1.11)	0.06
White Americans	18,144 (1,589)	1.00 (Ref.)	1.09 (0.93-1.27)	1.04 (0.86-1.25)	1.17 (0.94-1.47)	1.51 (1.06-2.14)	1.07 (1.01-1.14)	
**CHD**								
Black Americans	46,185 (1,480)	1.00 (Ref.)	0.96 (0.81-1.14)	1.23 (1.02-1.49)	1.25 (0.99-1.57)	1.56 (1.10-2.20)	1.09 (1.03-1.15)	0.61
White Americans	18,144 (736)	1.00 (Ref.)	1.05 (0.84-1.32)	1.06 (0.80-1.39)	1.15 (0.82-1.61)	1.38 (0.82-2.33)	1.12 (1.02-1.22)	
**Stroke**								
Black Americans	46,185 (701)	1.00 (Ref.)	0.96 (0.76-1.21)	0.91 (0.69-1.20)	1.03 (0.74-1.42)	1.18 (0.72-1.92)	1.12 (1.03-1.21)	0.23
White Americans	18,144 (204)	1.00 (Ref.)	1.12 (0.73-1.72)	1.14 (0.67-1.94)	1.63 (0.85-3.12)	1.20 (0.40-3.59)	1.02 (0.86-1.20)	
**Heart failure**								
Black Americans	46,185 (354)	1.00 (Ref.)	1.16 (0.84-1.59)	1.09 (0.73-1.62)	1.10 (0.67-1.80)	1.36 (0.63-2.92)	1.11 (0.98-1.26)	0.05
White Americans	18,144 (116)	1.00 (Ref.)	1.01 (0.56-1.79)	1.26 (0.61-2.59)	2.42 (1.01-5.79)	10.75 (2.94-39.39)	1.52 (1.18-1.97)	
**Cancer**								
Black Americans	46,185 (2,878)	1.00 (Ref.)	0.92 (0.82-1.04)	1.01 (0.89-1.16)	1.04 (0.89-1.22)	1.04 (0.82-1.33)	1.00 (0.96-1.03)	0.18
White Americans	18,144 (1,154)	1.00 (Ref.)	1.16 (0.96-1.39)	1.20 (0.96-1.49)	1.38 (1.06-1.81)	1.30 (0.85-1.99)	0.99 (0.93-1.06)	
**Other diseases**								
Black Americans	46,185 (4,444)	1.00 (Ref.)	1.13 (1.03-1.25)	1.14 (1.02-1.27)	1.20 (1.05-1.37)	1.32 (1.08-1.61)	1.04 (1.01-1.08)	0.58
White Americans	18,144 (2,263)	1.00 (Ref.)	1.03 (0.90-1.17)	0.96 (0.83-1.12)	1.02 (0.84-1.23)	0.82 (0.60-1.11)	0.96 (0.92-1.01)	

Abbreviation: mg/day, milligrams per day; no, number; CVD, cardiovascular disease; CHD, coronary heart disease; ref, reference.

a. Adjusted for age, race, sex, education, income, marital status, medical insurance, smoking, physical activity, alcohol consumption, body mass index, total energy intake, healthy eating index, and history of hypertension and CVD
b. Tested by the likelihood ratio test, comparing models with and without the multiplicative interaction term of sodium intake (continuous) x race

© 2024 Yoon H-S et al. *JAMA Network Open.*

eTable 6. All-Cause and Cause-Specific Mortality in Relation to Dietary Sodium Intake: Excluding First 2 Years of Follow-up [a]

Causes of death	No. of participants (no. of deaths)	Hazard Ratio (95% CI) by Dietary Sodium Intake (mg/day) [b] < 2,300	2,300-3,450	3,451-4,600	4,601-6,900	> 6,900	1,000 mg/day Increment	*P* Interaction [c]
**All-cause**								
Black Americans	45,170 (11,241)	1.00 (Ref.)	1.02 (0.96-1.08)	1.10 (1.03-1.18)	1.12 (1.03-1.22)	1.26 (1.11-1.43)	1.04 (1.02-1.06)	0.003
White Americans	17,649 (5,060)	1.00 (Ref.)	1.06 (0.98-1.16)	1.00 (0.90-1.11)	1.15 (1.01-1.31)	1.10 (0.90-1.34)	1.01 (0.98-1.04)	
**Total CVD**								
Black Americans	45,170 (3,740)	1.00 (Ref.)	0.98 (0.88-1.08)	1.10 (0.98-1.24)	1.10 (0.95-1.27)	1.34 (1.08-1.67)	1.07 (1.04-1.11)	0.006
White Americans	17,649 (1,447)	1.00 (Ref.)	1.07 (0.91-1.26)	1.01 (0.83-1.23)	1.17 (0.92-1.48)	1.41 (0.98-2.05)	1.06 (1.00-1.13)	
**CHD**								
Black Americans	45,170 (1,342)	1.00 (Ref.)	0.96 (0.81-1.15)	1.27 (1.04-1.55)	1.26 (0.99-1.60)	1.60 (1.11-2.30)	1.09 (1.03-1.15)	0.31
White Americans	17,649 (658)	1.00 (Ref.)	1.03 (0.81-1.32)	1.04 (0.78-1.39)	1.11 (0.78-1.58)	1.21 (0.69-2.11)	1.08 (0.98-1.19)	
**Stroke**								
Black Americans	45,170 (643)	1.00 (Ref.)	0.93 (0.73-1.18)	0.87 (0.65-1.16)	0.91 (0.65-1.28)	1.05 (0.63-1.77)	1.13 (1.04-1.23)	0.64
White Americans	17,649 (186)	1.00 (Ref.)	1.03 (0.65-1.61)	1.09 (0.63-1.90)	1.64 (0.83-3.22)	1.00 (0.31-3.21)	1.01 (0.85-1.21)	
**Heart failure**								
Black Americans	45,170 (334)	1.00 (Ref.)	1.14 (0.82-1.59)	1.09 (0.72-1.63)	1.11 (0.67-1.85)	1.31 (0.59-2.92)	1.11 (0.97-1.27)	0.02
White Americans	17,649 (113)	1.00 (Ref.)	1.00 (0.56-1.77)	1.16 (0.56-2.41)	2.26 (0.94-5.43)	10.72 (2.94-39.06)	1.58 (1.22-2.05)	
**Cancer**								
Black Americans	45,170 (2,669)	1.00 (Ref.)	0.91 (0.80-1.03)	1.02 (0.89-1.17)	1.03 (0.87-1.21)	1.06 (0.83-1.36)	1.00 (0.96-1.04)	0.14
White Americans	17,649 (1,054)	1.00 (Ref.)	1.19 (0.98-1.45)	1.26 (1.00-1.58)	1.47 (1.11-1.95)	1.44 (0.93-2.24)	1.00 (0.93-1.07)	
**Other diseases**								
Black Americans	45,170 (4,095)	1.00 (Ref.)	1.17 (1.05-1.29)	1.16 (1.03-1.30)	1.24 (1.08-1.42)	1.36 (1.10-1.67)	1.04 (1.01-1.08)	0.88
White Americans	17,649 (2,086)	1.00 (Ref.)	1.05 (0.92-1.19)	0.98 (0.83-1.15)	1.04 (0.85-1.27)	0.81 (0.58-1.12)	0.97 (0.92-1.02)	

Abbreviation: mg/day, milligrams per day; no, number; CVD, cardiovascular disease; CHD, coronary heart disease; ref, reference.

a. Excluded participants who died or were censored within the first two years of follow-up
b. Adjusted for age, race, sex, education, income, marital status, medical insurance, smoking, physical activity, alcohol consumption, body mass index, total energy intake, and healthy eating index
c. Tested by the likelihood ratio test, comparing models with and without the multiplicative interaction term of sodium intake (continuous) x race

© 2024 Yoon H-S et al. *JAMA Network Open.*

eTable 7. All-Cause and Cause-Specific Mortality in Relation to Dietary Sodium Intake: Excluding Individuals With History of Cardiovascular Disease

Causes of death	No. of participants (no. of deaths)	Hazard Ratio (95% CI) by Dietary Sodium Intake (mg/day) [a]					1,000 mg/day Increment	P Interaction [b]
		< 2,300	2,300-3,450	3,451-4,600	4,601-6,900	> 6,900		
**All-cause**								
Black Americans	41,178 (9,979)	1.00 (Ref.)	1.02 (0.95-1.08)	1.09 (1.01-1.17)	1.08 (0.99-1.18)	1.20 (1.05-1.36)	1.03 (1.01-1.05)	<0.001
White Americans	15,321 (4,128)	1.00 (Ref.)	1.01 (0.91-1.11)	0.95 (0.85-1.07)	1.06 (0.92-1.22)	1.00 (0.81-1.24)	1.01 (0.97-1.04)	
**Total CVD**								
Black Americans	41,178 (3,106)	1.00 (Ref.)	1.01 (0.90-1.13)	1.13 (0.99-1.29)	1.12 (0.96-1.31)	1.36 (1.07-1.73)	1.06 (1.03-1.10)	0.003
White Americans	15,321 (1,015)	1.00 (Ref.)	0.99 (0.82-1.21)	0.97 (0.77-1.22)	1.02 (0.77-1.35)	1.16 (0.75-1.80)	1.07 (1.00-1.15)	
**CHD**								
Black Americans	41,178 (1,074)	1.00 (Ref.)	1.04 (0.85-1.27)	1.33 (1.06-1.67)	1.33 (1.02-1.74)	1.67 (1.12-2.49)	1.08 (1.01-1.15)	0.07
White Americans	15,321 (436)	1.00 (Ref.)	0.82 (0.61-1.11)	0.96 (0.68-1.36)	0.82 (0.54-1.26)	0.87 (0.45-1.68)	1.11 (0.99-1.24)	
**Stroke**								
Black Americans	41,178 (501)	1.00 (Ref.)	0.86 (0.65-1.14)	0.84 (0.60-1.16)	0.81 (0.55-1.18)	0.99 (0.56-1.74)	1.10 (1.01-1.21)	0.36
White Americans	15,321 (137)	1.00 (Ref.)	0.98 (0.58-1.64)	1.01 (0.53-1.91)	1.43 (0.65-3.19)	1.02 (0.26-3.99)	1.04 (0.84-1.29)	
**Heart failure**								
Black Americans	41,178 (263)	1.00 (Ref.)	1.27 (0.87-1.86)	1.16 (0.73-1.85)	1.24 (0.70-2.20)	1.42 (0.58-3.48)	1.12 (0.97-1.30)	0.28
White Americans	15,321 (64)	1.00 (Ref.)	1.18 (0.54-2.56)	1.65 (0.62-4.43)	4.76 (1.44-15.69)	22.96 (3.78-139.3)	1.54 (1.08-2.20)	
**Cancer**								
Black Americans	41,178 (2,556)	1.00 (Ref.)	0.89 (0.79-1.01)	0.97 (0.84-1.12)	0.98 (0.83-1.16)	1.03 (0.80-1.33)	1.00 (0.96-1.04)	0.08
White Americans	15,321 (926)	1.00 (Ref.)	1.10 (0.90-1.34)	1.10 (0.86-1.40)	1.35 (1.01-1.82)	1.39 (0.87-2.22)	1.00 (0.93-1.08)	
**Other diseases**								
Black Americans	41,178 (3,624)	1.00 (Ref.)	1.15 (1.03-1.28)	1.16 (1.02-1.31)	1.19 (1.03-1.37)	1.29 (1.03-1.60)	1.03 (1.00-1.07)	0.34
White Americans	15,321 (1,708)	1.00 (Ref.)	1.00 (0.86-1.16)	0.95 (0.80-1.13)	0.98 (0.79-1.22)	0.74 (0.52-1.05)	0.96 (0.91-1.02)	

Abbreviation: mg/day, milligrams per day; no, number; CVD, cardiovascular disease; CHD, coronary heart disease; ref, reference.

a. Adjusted for age, race, sex, education, income, marital status, medical insurance, smoking, physical activity, alcohol consumption, body mass index, total energy intake, and healthy eating index

b. Tested by the likelihood ratio test, comparing models with and without the multiplicative interaction term of sodium intake (continuous) x race

© 2024 Yoon H-S et al. *JAMA Network Open.*

eTable 8. All-Cause and Cause-Specific Mortality in Relation to Dietary Sodium Intake: Further Adjusting for Comorbidity Index

Causes of death	No. of participants (no. of deaths)	Hazard Ratio (95% CI) by Dietary Sodium Intake (mg/day) [a] < 2,300	2,300-3,450	3,451-4,600	4,601-6,900	> 6,900	1,000 mg/day Increment	*P* Interaction [b]
**All-cause**								
Black Americans	46,185 (12,256)	1.00 (Ref.)	1.01 (0.95-1.07)	1.07 (1.00-1.14)	1.08 (1.00-1.17)	1.20 (1.06-1.35)	1.03 (1.01-1.05)	0.002
White Americans	18,144 (5,555)	1.00 (Ref.)	1.05 (0.97-1.14)	0.97 (0.88-1.07)	1.09 (0.96-1.23)	1.06 (0.88-1.28)	1.00 (0.97-1.03)	
**Total CVD**								
Black Americans	46,185 (4,112)	1.00 (Ref.)	0.99 (0.89-1.09)	1.09 (0.97-1.22)	1.09 (0.95-1.25)	1.31 (1.06-1.61)	1.06 (1.03-1.10)	0.02
White Americans	18,144 (1,589)	1.00 (Ref.)	1.08 (0.93-1.26)	1.01 (0.84-1.21)	1.13 (0.90-1.41)	1.41 (0.99-2.00)	1.06 (1.00-1.13)	
**CHD**								
Black Americans	46,185 (1,480)	1.00 (Ref.)	0.95 (0.80-1.12)	1.20 (0.99-1.44)	1.20 (0.95-1.50)	1.47 (1.04-2.07)	1.08 (1.02-1.14)	0.45
White Americans	18,144 (736)	1.00 (Ref.)	1.04 (0.83-1.31)	1.02 (0.77-1.34)	1.10 (0.79-1.54)	1.27 (0.75-2.15)	1.11 (1.01-1.21)	
**Stroke**								
Black Americans	46,185 (701)	1.00 (Ref.)	0.94 (0.74-1.19)	0.87 (0.66-1.15)	0.98 (0.71-1.35)	1.09 (0.67-1.78)	1.11 (1.02-1.20)	0.31
White Americans	18,144 (204)	1.00 (Ref.)	1.11 (0.73-1.71)	1.10 (0.65-1.87)	1.55 (0.81-2.96)	1.10 (0.36-3.30)	1.00 (0.85-1.19)	
**Heart failure**								
Black Americans	46,185 (354)	1.00 (Ref.)	1.14 (0.83-1.57)	1.05 (0.71-1.56)	1.04 (0.64-1.71)	1.27 (0.59-2.73)	1.10 (0.97-1.25)	0.04
White Americans	18,144 (116)	1.00 (Ref.)	0.99 (0.56-1.76)	1.19 (0.58-2.44)	2.27 (0.96-5.38)	9.93 (2.74-36.0)	1.50 (1.16-1.94)	
**Cancer**								
Black Americans	46,185 (2,878)	1.00 (Ref.)	0.92 (0.82-1.04)	1.01 (0.89-1.16)	1.04 (0.89-1.22)	1.05 (0.82-1.33)	1.00 (0.96-1.03)	0.21
White Americans	18,144 (1,154)	1.00 (Ref.)	1.15 (0.96-1.39)	1.18 (0.95-1.46)	1.35 (1.03-1.76)	1.27 (0.83-1.94)	0.99 (0.92-1.05)	
**Other diseases**								
Black Americans	46,185 (4,444)	1.00 (Ref.)	1.12 (1.01-1.23)	1.10 (0.99-1.23)	1.15 (1.01-1.32)	1.26 (1.03-1.54)	1.04 (1.00-1.07)	0.59
White Americans	18,144 (2,263)	1.00 (Ref.)	1.02 (0.90-1.16)	0.94 (0.81-1.10)	0.98 (0.81-1.19)	0.78 (0.57-1.06)	0.96 (0.91-1.01)	

Abbreviation: mg/day, milligrams per day; no, number; CVD, cardiovascular disease; CHD, coronary heart disease; ref, reference.

a. Adjusted for age, race, sex, education, income, marital status, medical insurance, smoking, physical activity, alcohol consumption, body mass index, total energy intake, healthy eating index, and comorbidity index
b. Tested by the likelihood ratio test, comparing models with and without the multiplicative interaction term of sodium intake (continuous) x race

© 2024 Yoon H-S et al. *JAMA Network Open.*

eTable 9. All-Cause and Cause-Specific Mortality in Relation to Dietary Sodium Intake: Competing Risk Analysis [a]

Causes of death	No. of participants (no. of deaths)	Hazard Ratio (95% CI) by Dietary Sodium Intake (mg/day) [b] < 2,300	2,300-3,450	3,451-4,600	4,601-6,900	> 6,900	1,000 mg/day Increment	*P* Interaction [c]
**All-cause**								
Black Americans	46,185 (12,256)	1.00 (Ref.)	1.02 (0.96-1.08)	1.08 (1.01-1.15)	1.10 (1.01-1.19)	1.21 (1.07-1.36)	1.03 (1.01-1.05)	<0.001
White Americans	18,144 (5,555)	1.00 (Ref.)	1.06 (0.97-1.15)	0.98 (0.89-1.09)	1.13 (1.00-1.27)	1.09 (0.91-1.32)	1.01 (0.98-1.04)	
**Total CVD**								
Black Americans	46,185 (4,112)	1.00 (Ref.)	0.99 (0.90-1.10)	1.08 (0.96-1.20)	1.08 (0.94-1.24)	1.26 (1.03-1.56)	1.06 (1.03-1.10)	0.02
White Americans	18,144 (1,589)	1.00 (Ref.)	1.09 (0.93-1.27)	1.05 (0.87-1.27)	1.17 (0.93-1.46)	1.52 (1.08-2.16)	1.08 (1.02-1.15)	
**CHD**								
Black Americans	46,185 (1,480)	1.00 (Ref.)	0.96 (0.81-1.13)	1.18 (0.98-1.43)	1.19 (0.95-1.49)	1.41 (1.00-2.00)	1.07 (1.01-1.13)	0.50
White Americans	18,144 (736)	1.00 (Ref.)	1.04 (0.83-1.31)	1.06 (0.80-1.39)	1.14 (0.82-1.58)	1.36 (0.81-2.27)	1.13 (1.03-1.24)	
**Stroke**								
Black Americans	46,185 (701)	1.00 (Ref.)	0.95 (0.75-1.20)	0.85 (0.64-1.13)	0.96 (0.69-1.34)	1.04 (0.64-1.71)	1.10 (1.01-1.20)	0.25
White Americans	18,144 (204)	1.00 (Ref.)	1.09 (0.72-1.66)	1.14 (0.68-1.91)	1.57 (0.83-2.98)	1.13 (0.35-3.66)	1.02 (0.86-1.21)	
**Heart failure**								
Black Americans	46,185 (354)	1.00 (Ref.)	1.16 (0.84-1.58)	1.04 (0.70-1.53)	1.03 (0.64-1.66)	1.21 (0.58-2.52)	1.09 (0.96-1.25)	0.03
White Americans	18,144 (116)	1.00 (Ref.)	0.98 (0.54-1.76)	1.27 (0.60-2.67)	2.40 (1.01-5.71)	11.2 (3.00-41.6)	1.53 (1.27-1.85)	
**Cancer**								
Black Americans	46,185 (2,878)	1.00 (Ref.)	0.92 (0.81-1.04)	1.00 (0.87-1.15)	1.03 (0.88-1.21)	1.01 (0.80-1.29)	0.99 (0.96-1.03)	0.33
White Americans	18,144 (1,154)	1.00 (Ref.)	1.14 (0.95-1.38)	1.21 (0.97-1.51)	1.36 (1.04-1.79)	1.30 (0.83-2.03)	0.99 (0.93-1.06)	
**Other diseases**								
Black Americans	46,185 (4,444)	1.00 (Ref.)	1.14 (1.03-1.25)	1.10 (0.98-1.23)	1.15 (1.01-1.32)	1.24 (1.01-1.52)	1.04 (1.00-1.07)	0.67
White Americans	18,144 (2,263)	1.00 (Ref.)	1.01 (0.89-1.15)	0.96 (0.83-1.12)	0.99 (0.82-1.19)	0.78 (0.58-1.06)	0.96 (0.92-1.01)	

Abbreviation: mg/day, milligrams per day; no, number; CVD, cardiovascular disease; CHD, coronary heart disease; ref, reference.

a. For cause-specific mortality outcomes
b. Adjusted for age, race, sex, education, income, marital status, medical insurance, smoking, physical activity, alcohol consumption, body mass index, total energy intake, and healthy eating index
c. Tested by the likelihood ratio test, comparing models with and without the multiplicative interaction term of sodium intake (continuous) x race

© 2024 Yoon H-S et al. *JAMA Network Open.*

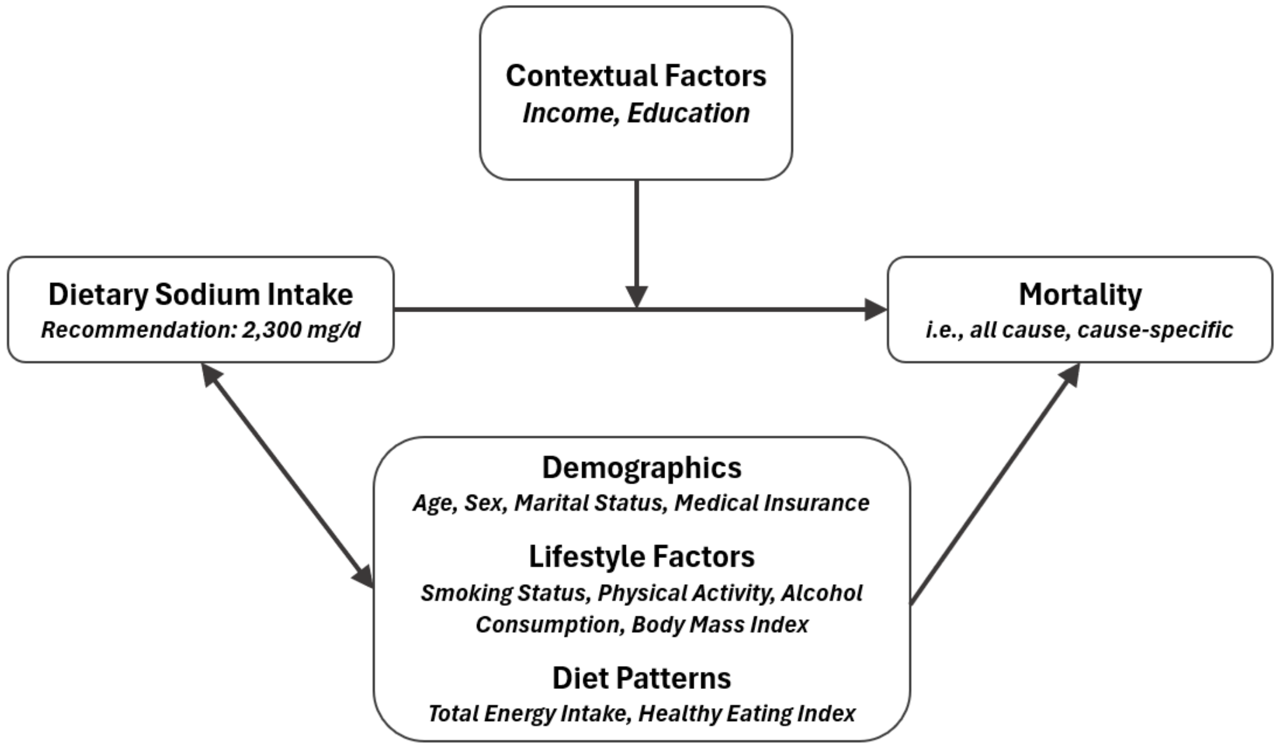

eFigure. Conceptual Framework for Research Questions

© 2024 Yoon H-S et al. *JAMA Network Open.*
