# Supplementary material for: Sodium Intake and Cause-Specific Mortality Among Predominantly Low-Income Black and White US Residents
Source: JAMA Netw Open. 2024 Mar 26;7(3):e243802. doi: 10.1001/jamanetworkopen.2024.3802 (PMC10966417; doi:10.1001/jamanetworkopen.2024.3802)
Supplement: Supplement 2. — Data Sharing Statement [file jamanetwopen-e243802-s002.pdf]

Data Sharing Statement

Yoon. Sodium Intake and Cause-Specific Mortality Among Predominantly Low-Income Black and White Americans. *JAMA Netw Open*. Published March 26, 2024.
doi:10.1001/jamanetworkopen.2024.3802

Data
Data available: Yes
Data types: Data dictionary
How to access data: Data for the study is available via a formal application to the parent study
When available: With publication

Supporting Documents
Document types: None

Additional Information
Who can access the data: Anyone requesting the data
Types of analyses: For any purpose
Mechanisms of data availability: After an approved application to the parent study
